# Incidental Diagnosis of Honeycomb Gallbladder on Point-of-Care Ultrasound

**DOI:** 10.7759/cureus.32650

**Published:** 2022-12-17

**Authors:** Ridhima Ghei, Leland Perice, Kay Odashima

**Affiliations:** 1 Emergency Medicine, Maimonides Medical Center, Brooklyn, USA

**Keywords:** pocus (point of care ultrasound), point-of-care-ultrasound, gallbladder disease, gallbladder disorders, congenital gallbladder abnormalities, gallbladder anomaly

## Abstract

Honeycomb gallbladder is a term that has been coined to describe a multiseptated gallbladder. In this case report, we describe a pregnant patient who had an extensive work-up of her abdominal pain and was found to have this incidental finding.

## Introduction

Multiseptated gallbladder (MSG) is a rare anatomical finding. This condition was first described in 1952, with only about 150 documented cases worldwide [1]. Presentations of MSG can vary from pediatric to adult patients and asymptomatic to symptomatic patients. Multiple theories have been devised about the pathogenesis of MSG, but given the limited number of cases, there are no definitive guidelines on its management [2,3]. MSG can present with abdominal pain, and of those with pain, many have symptom resolution following cholecystectomy [3]. Yet, whether surgery needs to be done emergently is not established. Currently, medical or surgical management varies on a case-by-case basis depending on work-up findings and a patient’s symptoms. It is overall considered a benign disorder but can be associated with other biliary abnormalities, so an outpatient work-up is warranted [1].

Here, we present a case describing a pregnant patient at an urban teaching hospital in New York City who presented to the emergency department (ED) with a chief complaint of abdominal pain. She received an extensive work-up to rule out any emergent pathologies and was ultimately diagnosed with MSG. This case illustrates the benign nature of this condition and supports the practice that this finding alone does not require further work-up in the ED.

## Case presentation

The patient was a 28-year-old female, gravida 6 para 3, who initially presented with abdominal pain. The pain started approximately 6 hours prior to presentation and was sudden in onset. Her pain was localized to the lower abdomen but was worse on the right compared to the left side. A home pregnancy test was positive one week prior. She denied fever, nausea, vomiting, urinary symptoms, vaginal bleeding, vaginal discharge, constipation, and diarrhea.

The patient’s initial vital signs were a temperature of 98.7°F (37°C), a heart rate of 92 beats/min, a respiratory rate of 16 breaths/min, a blood pressure of 128/65 mmHg, and an oxygen saturation of 100% on room air. On physical exam, her abdomen was soft, non-distended, and non-tender to palpation in all quadrants. The serum beta-hcg level was >138,000 mIU/mL. The remainder of her laboratory results were within normal limits. A point-of-care transabdominal pelvic ultrasound was performed by the emergency physician, which showed a live intrauterine pregnancy with a normal fetal heart rate, thereby making the diagnosis of ectopic pregnancy highly unlikely. Both of her ovaries were normal in size with Doppler arterial and venous flow present, making ovarian torsion unlikely. 

**Figure 1:**
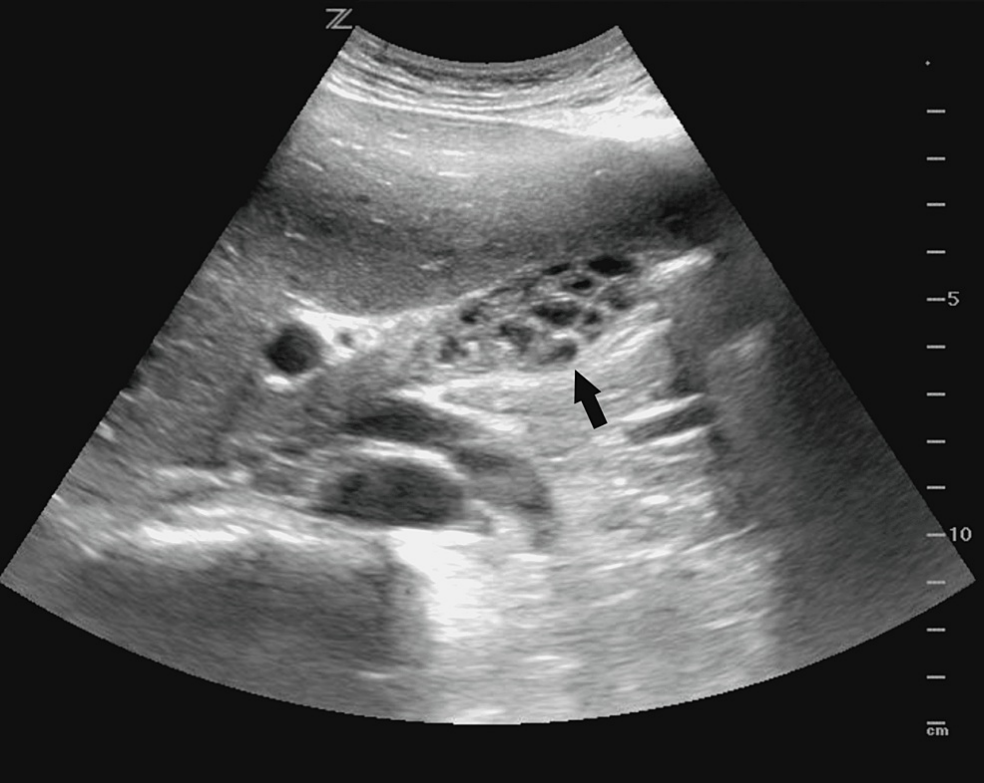
Multiseptated gallbladder (black arrow) seen on ultrasound taken in the emergency department. Multiple septations are noted on the point-of-care ultrasound performed initially in the emergency department.

Other possibilities of abdominal pain were sought, and a right upper quadrant point-of-care ultrasound showed an abnormal gallbladder with multiple septations (Figure 1). The common bile duct (CBD) was not dilated, sonographic Murphy’s sign was negative, the gallbladder wall was not thickened, and there was no pericholecystic fluid.

**Figure 2:**
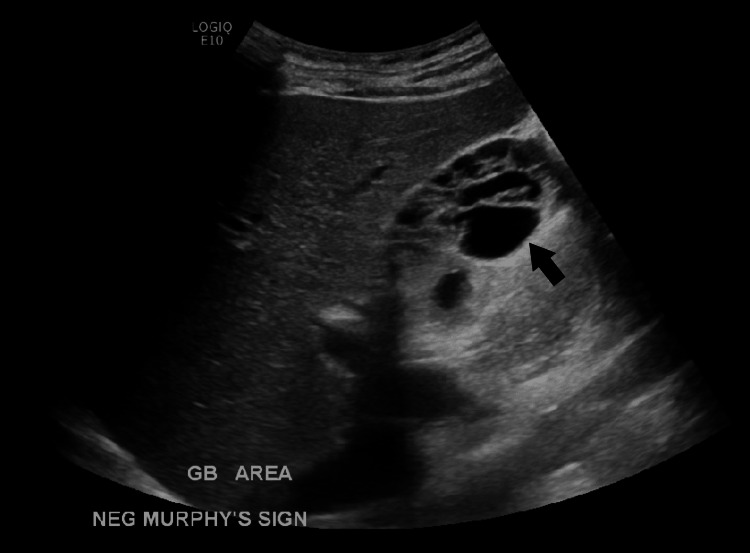
Multiseptated gallbladder (black arrow) seen on ultrasound taken in the radiology department. This is the long-axis view of the multiseptated gallbladder which was obtained by the radiology department. GB: Gallbladder

**Figure 3:**
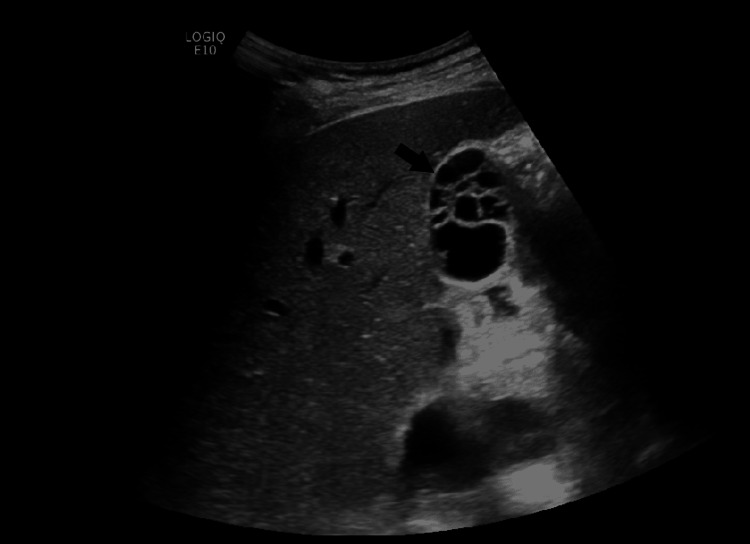
Multiseptated gallbladder (black arrow) seen on ultrasound taken in the radiology department. This is a short-axis view of the multiseptated gallbladder which was obtained by the radiology department.

Subsequently, a radiology-performed right upper quadrant ultrasound showed a normal hepatic echotexture and architecture. No focal lesions were identified. There was no intra- or extrahepatic bile duct dilatation. The proximal CBD measured 2.5 mm. There was no sonographic Murphy's sign or visible stones. The gallbladder appeared multiseptated with multiple cystic spaces (Figure 2 and Figure 3). There was no gallbladder wall thickening or pericholecystic fluid. The hepatic veins were unremarkable. There was no pancreatic ductal dilation.

**Figure 4:**
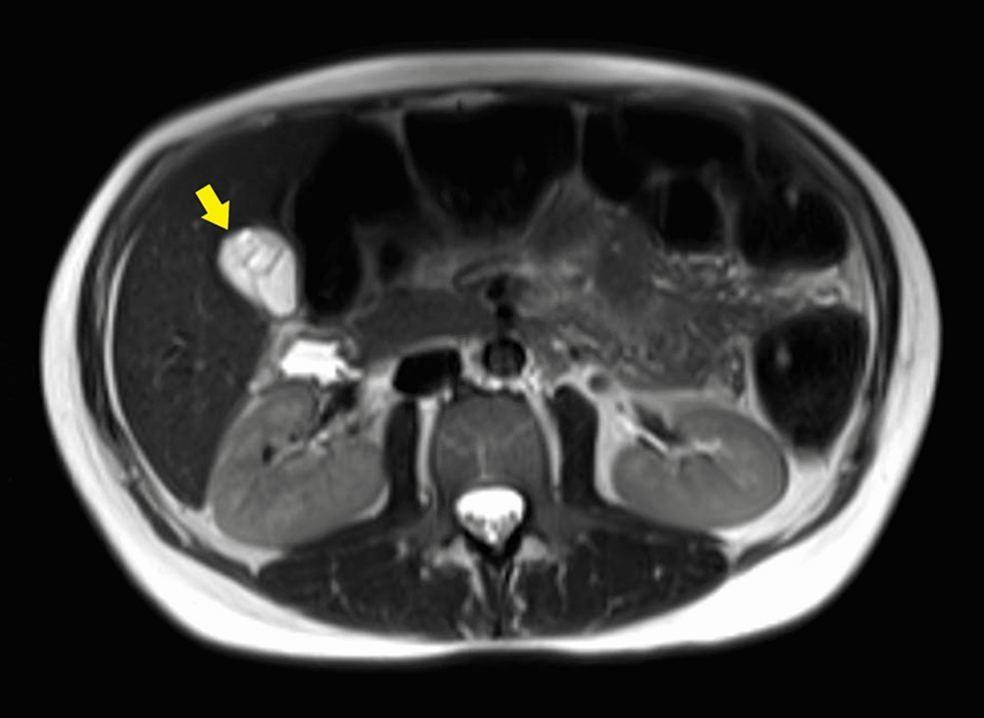
Septations seen in gallbladder on MRI abdomen/pelvis (yellow arrow). MRI abdomen/pelvis without IV contrast was obtained to rule out other abdominal pathologies and also demonstrates septations in the gallbladder. MRI: Magnetic resonance imaging

Magnetic resonance imaging of the abdomen and pelvis was performed without IV contrast to rule out appendicitis, which reaffirmed many of the ultrasound findings. The gallbladder appeared multiseptated with milk of calcium bile (Figure 4). There were no signs of cholecystitis. The bile ducts appeared normal. The other organs, including the appendix, were within normal limits. There were a few follicles noted in the right ovary and trace free fluid in the right lower quadrant and pelvis. A definitive etiology of the patient’s abdominal pain was not found, and she was discharged home.

## Discussion

MSG, or honeycomb gallbladder, is a rare finding with multiple theories behind its pathogenesis. One theory is that there is incomplete cavitation of the gallbladder in its solid embryologic form [4-6]. The “wrinkling” and “Phrygian cap” theories suggest that the gallbladder may develop faster than its surrounding structures and subsequently wrinkle due to lack of space. This wrinkling may result in invaginations of the gallbladder which then merge with intraepithelial tissue [2,3]. Histologically, MSGs have been found to have epithelial layers intermingled with a muscular layer [1,3,7].

Due to the limited number of cases reported globally, there are currently no established guidelines on the evaluation and management of MSG. The majority of cases of MSG have been diagnosed on ultrasound; however, this diagnosis has also been made using magnetic resonance cholangiopancreatography and computed tomography [1,3,7]. In patients with MSG, it is prudent to rule out other biliary tract abnormalities, particularly those that are at risk for malignant transformation [1]. Hsieh et al. described three cases of MSG with concurrent choledochal cysts who underwent excision of the biliary tree combined with hepaticojejunostomy, choledochoduodenostomy, or Roux-en-Y anastomosis [3].

If a patient is asymptomatic and does not have associated biliary tract abnormalities, outpatient follow-up is sufficient [5,6]. The benefit of performing elective surgeries for patients with MSG is unclear. Based on a case reported by Hsieh et al., a patient who had undergone cholecystectomy had symptoms persist after the surgery, indicating that MSG was not the cause of the pain [3]. Additionally, patients with MSG who have gastrointestinal symptoms often recover without treatment [3]. Therefore, after ruling out any associated biliary tract anomalies, it may be best to pursue conservative management and address other etiologies of biliary symptoms before a cholecystectomy [3].

In the case presented here, the patient’s symptoms resolved during her ED visit. It is unclear what caused the patient’s pain. Emergent and surgical causes of the patient’s abdominal pain had been ruled out through multiple imaging modalities. The patient had improvement of her symptoms during the ED visit and was safely discharged home with diagnoses of abdominal pain and MSG. She was referred to outpatient gastroenterology for follow-up. She had no further visits to our hospital until her delivery seven months later, which was uneventful per chart review.

## Conclusions

MSG is a rare anatomical finding of likely minimal clinical significance in the ED. It is often a benign finding and, provided there are no other biliary tree abnormalities, even symptomatic patients can often recover without treatment. For the patient in this case report, MSG was an incidental finding, and she did not require any emergency intervention for it.
